# Local Anesthetics Induce Apoptosis in Human Thyroid Cancer Cells through the Mitogen-Activated Protein Kinase Pathway

**DOI:** 10.1371/journal.pone.0089563

**Published:** 2014-02-21

**Authors:** Yuan-Ching Chang, Yi-Chiung Hsu, Chien-Liang Liu, Shih-Yuan Huang, Meng-Chun Hu, Shih-Ping Cheng

**Affiliations:** 1 Graduate Institute of Physiology, National Taiwan University College of Medicine, Taipei, Taiwan; 2 Department of Surgery, Mackay Memorial Hospital and Mackay Medical College, Taipei, Taiwan; 3 Institute of Statistical Science, Academia Sinica, Taipei, Taiwan; 4 Mackay Junior College of Medicine, Nursing, and Management, Taipei, Taiwan; 5 Graduate Institute of Medical Sciences, Department of Pharmacology, Taipei Medical University, Taipei, Taiwan; Southern Medical University, China

## Abstract

Local anesthetics are frequently used in fine-needle aspiration of thyroid lesions and locoregional control of persistent or recurrent thyroid cancer. Recent evidence suggests that local anesthetics have a broad spectrum of effects including inhibition of cell proliferation and induction of apoptosis in neuronal and other types of cells. In this study, we demonstrated that treatment with lidocaine and bupivacaine resulted in decreased cell viability and colony formation of both 8505C and K1 cells in a dose-dependent manner. Lidocaine and bupivacaine induced apoptosis, and necrosis in high concentrations, as determined by flow cytometry. Lidocaine and bupivacaine caused disruption of mitochondrial membrane potential and release of cytochrome c, accompanied by activation of caspase 3 and 7, PARP cleavage, and induction of a higher ratio of Bax/Bcl-2. Based on microarray and pathway analysis, apoptosis is the prominent transcriptional change common to lidocaine and bupivacaine treatment. Furthermore, lidocaine and bupivacaine attenuated extracellular signal-regulated kinase 1/2 (ERK1/2) activity and induced activation of p38 mitogen-activated protein kinase (MAPK) and c-jun N-terminal kinase. Pharmacological inhibitors of MAPK/ERK kinase and p38 MAPK suppressed caspase 3 activation and PARP cleavage. Taken together, our results for the first time demonstrate the cytotoxic effects of local anesthetics on thyroid cancer cells and implicate the MAPK pathways as an important mechanism. Our findings have potential clinical relevance in that the use of local anesthetics may confer previously unrecognized benefits in the management of patients with thyroid cancer.

## Introduction

Thyroid cancer is the most common of all endocrine cancers, and the incidence of thyroid cancer is increasing worldwide [1], [2]. The majority of thyroid cancers are well differentiated with papillary and follicular thyroid carcinoma being the most common types. Although well-differentiated thyroid cancer has a generally favorable prognosis, the overall recurrence rates could be as high as 35% [3]. Persistent or recurrent disease occurs largely in the neck. Moreover, development of recurrence is associated with a higher mortality. Thirty-year cancer mortality rates were reported to be about 12% in patients with local recurrence and 43% in those with distant recurrence [3].

The treatment options for persistent or recurrent disease include additional surgery and radioactive iodine therapy. Compartmental neck dissection for locoregional recurrence is recommended by the American Thyroid Association guidelines [4]. Another alternative treatment with promising results is ultrasound-guided percutaneous ethanol injection and radiofrequency ablation [5]–[7]. During the procedure, lidocaine infiltration to the puncture site and soft tissue around the recurrent tumor is a simple and effective method of pain control [8]. No adverse effects were reported except for that ultrasound image quality may be compromised with a large amount of lidocaine [7].

In addition to the well established action of analgesia and antiarrhythmia, studies have demonstrated that local anesthetics have a broad spectrum of effects including anti-inflammatory and antimicrobial properties [9]. Recent observations of their chondrotoxicity call for caution in the clinical use of intra-articular injections [10]. Furthermore, there is growing evidence that local anesthetics might exert beneficial actions in the treatment of cancer by inhibition of cell proliferation, invasion, and migration [11]–[13]. These effects appear unrelated to their modulation of sodium channels [14]. In this regard, we have shown that apoptosis plays an important role in the local anesthetic-induced cell toxicity of breast cancer cells [15]. Previous studies suggest that neuronal apoptosis induced by local anesthetics is mediated, at least in part, by mitogen-activated protein kinase (MAPK) pathway [16], [17]. However, little is known whether similar mechanisms apply to local anesthetic-induced cytotoxicity in cancer cells.

Given that local anesthetics are frequently used in fine-needle aspiration of thyroid lesions and locoregional control of persistent or recurrent thyroid cancer, it would be interesting to find out whether local anesthetics have an antiproliferative effect on thyroid cancer cells. We reported here that commonly used local anesthetics, lidocaine and bupivacaine, inhibited cell growth and induced apoptosis of human thyroid cancer cells in clinically relevant concentrations. Furthermore, in agreement with previous studies and our microarray and pathway analysis, we observed that MAPK signaling pathways were involved.

## Results

### Inhibition of cell growth and colony formation by local anesthetics

**Figure 1:**
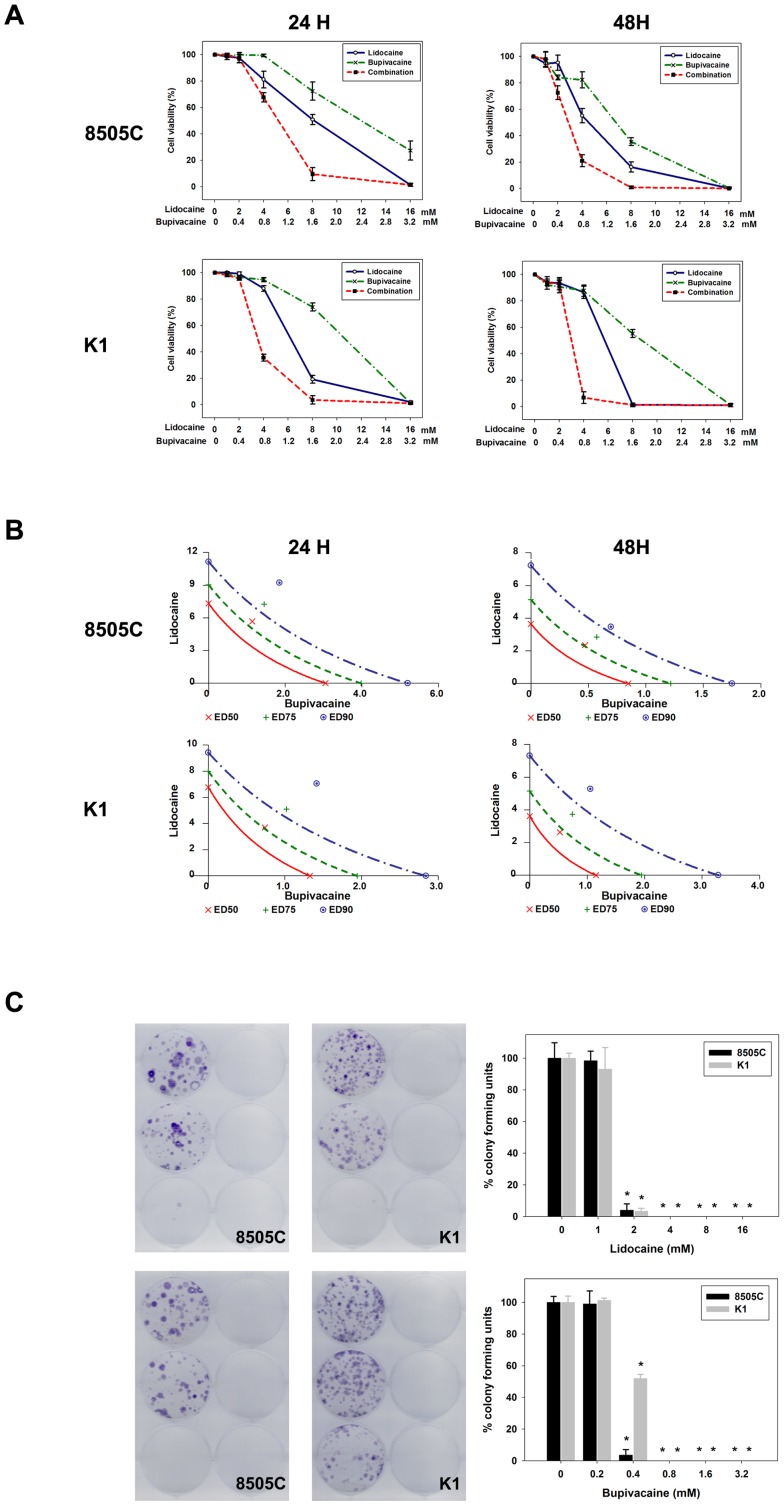
Effects of local anesthetics on cell growth and colony formation of human thyroid cancer cells. (**A**) 8505C and K1 cells were treated with serial dilutions of lidocaine and bupivacaine, individually or in combinations, for 24 and 48 h. Error bars represent standard error of the mean. (**B**) A conservative isobologram demonstrates that lidocaine and bupivacaine acts antagonistically to inhibit the cell growth of thyroid cancer cells. ED indicates effective dose. The ED50 (red X), ED75 (green crosses), and ED90 (blue circles) are graphed. (**C**) Treatment with lidocaine and bupivacaine resulted in reduction of colony formation of the 8505C and K1 cell lines. Error bars represent standard error of the mean. *, P<0.01 *versus* control.

Cell viability of 8505C and K1 thyroid cancer cells was determined by incubating with lidocaine and bupivacaine at serially diluted concentrations for 24 and 48 hours. Lidocaine and bupivacaine inhibited the growth of both thyroid cancer cells in a dose-dependent manner (Fig. 1A). At 24 hours, the median effective dose (ED50) of lidocaine was 7.3 mM for 8505C cells and 6.8 mM for K1 cells, respectively. These were lower than the commonly used concentration of 1% (w/v) lidocaine (42.67 mM). Similarly, the ED50 at 24 hours of bupivacaine was 3.1 mM for 8505C cells and 1.3 mM for K1 cells. Both were much lower than the clinical concentration of 0.5% (w/v) bupivacaine (17.34 mM).

To examine whether the cytotoxic effect is restricted to amide-type local anesthetics, thyroid cancer cells were also treated with an ester-type local anesthetics, procaine. The ED50 was 39.6 mM and 30.9 mM, respectively, suggesting that the effect correlated with the potency, but not the class, of local anesthetics. Furthermore, to exclude the possibility that changes in pH or osmolality would account for the cytotoxic effects, pH and osmolality of the culture media for experiments were determined. There were no significant variations in pH or osmolality (Fig. S1), indicating that the observed effects were pharmacological in nature.

Drug synergy was determined by the combination index (CI) and isobologram analyses according to the median effect methods (Fig. 1B). The CI was 1.17±0.03 for 8505C cells and 1.14±0.06 for K1 cells. These results suggest that combination of lidocaine and bupivacaine yielded slight antagonism.

Furthermore, the effects of local anesthetics on tumor cell growth were determined by clonogenic assay. The results are shown in Fig. 1C. A significant dose-dependent reduction in colonies was observed in 8505C and K1 cells. Thyroid cancer cells did not form colonies following treatment with lidocaine >4 mM or bupivacaine >0.8 mM.

### Induction of apoptosis by local anesthetics

**Figure 2:**
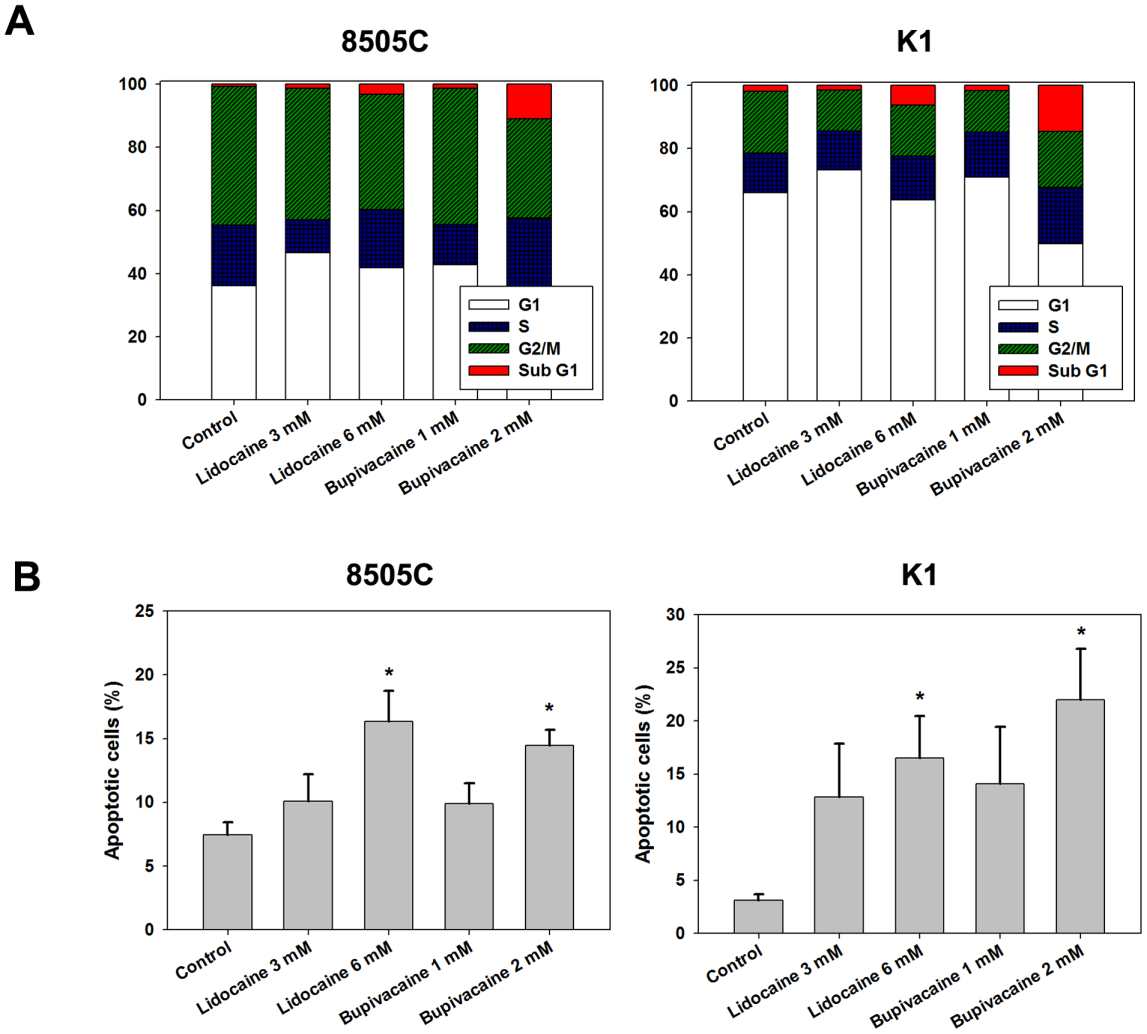
Effects of local anesthetics on cell cycle and apoptosis in human thyroid cancer cells. (**A**) 8505C and K1 cells were treated with the indicated concentrations of lidocaine and bupivacaine for 24 h. Thereafter, the cells were washed, fixed, and stained with propidium iodide (PI) and were analyzed for DNA content in different phases of the cell cycle by flow cytometry. (**B**) Thyroid cancer cells were treated with lidocaine and bupivacaine for 48 h, and cells were subsequently stained with fluorescein-conjugated annexin V and PI and analyzed by flow cytometry. Error bars represent standard error of the mean. *, P<0.05 *versus* control.

To determine the basis of reduced cell viability, cell cycle analysis was performed. There was no cell cycle arrest in thyroid cancer cells treated with lidocaine and bupivacaine for 6, 24, and 48 hours (data not shown). However, we observed a dose-dependent increase in the sub-G1 (hypodiploid) fraction following treatment with lidocaine and bupivacaine (Fig. 2A). We also used annexin V/propidium iodide (PI) dual staining for further confirmation of local anesthetics-induced apoptosis in thyroid cancer cells. As shown in Fig. 2B, treatment with lidocaine and bupivacaine resulted in apoptosis in a dose-dependent manner. Necrosis was also observed in high concentrations of lidocaine and bupivacaine. Taken together, these results suggest that growth suppression by local anesthetics in thyroid cancer cells involves induction of apoptosis.

### Assessment of mitochondrial dysfunction

**Figure 3:**
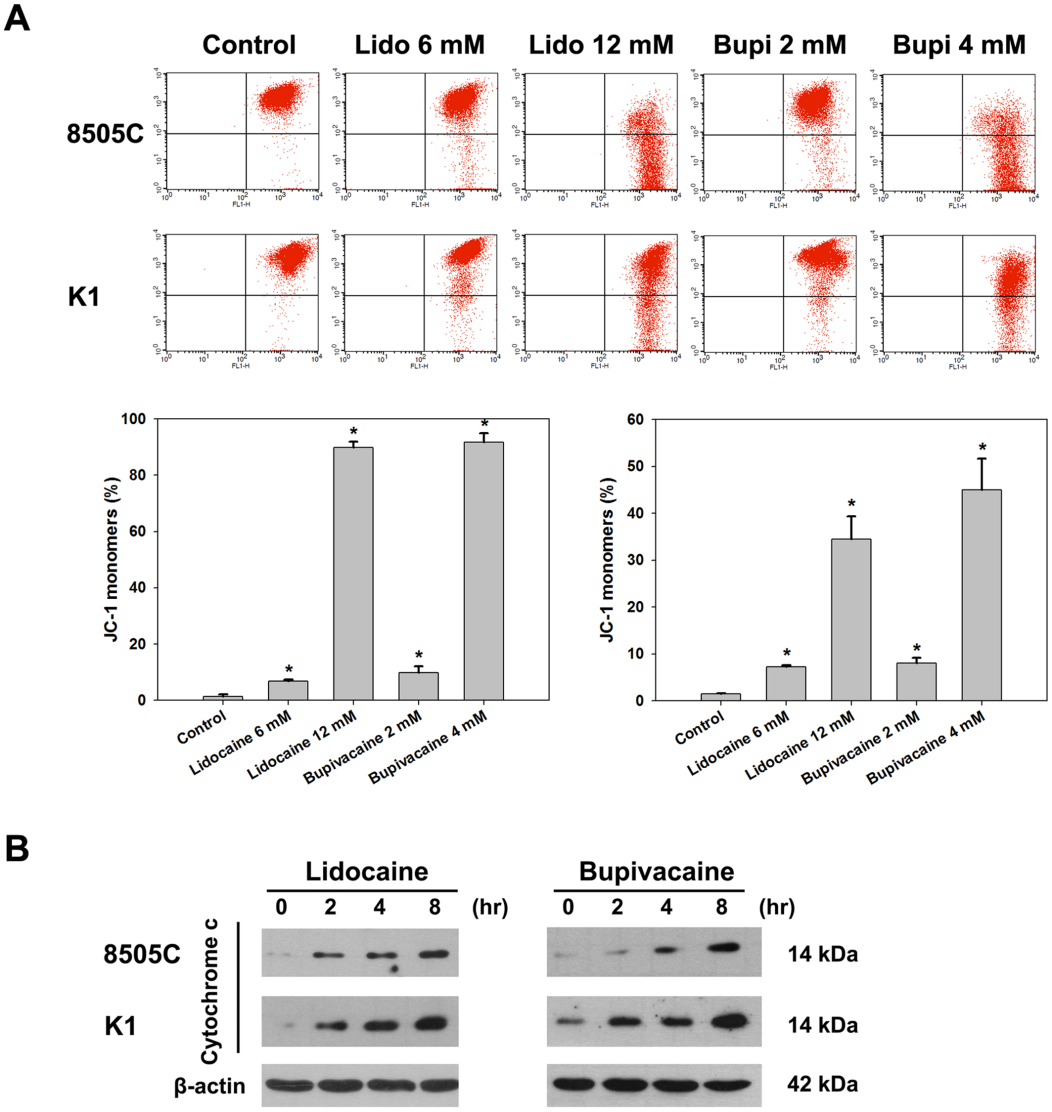
Change in mitochondrial membrane potential (ΔΨ_m_) and cytochrome c release induced by local anesthetics in human thyroid cancer cells. (**A**) 8505C and K1 cells were treated with the indicated concentrations of lidocaine and bupivacaine for 16 h. ΔΨ_m_ change was monitored by loading with JC-1 and was analyzed by flow cytometry. Error bars represent standard error of the mean. *, P<0.05 *versus* control. (**B**) Thyroid cancer cells were treated with lidocaine (6 mM) and bupivacaine (2 mM) for the indicated periods of time. Cytochrome c release from mitochondria to cytosol was determined by Western blotting. The blots were stripped and reprobed with an antibody against actin for equal loading.

A decrease in mitochondrial membrane potential is one of the earliest events in apoptosis. Mitochondrial membrane integrity was evaluated using the cationic dye JC-1, a highly specific probe for detecting changes in mitochondrial ΔΨ_m_. JC-1 forms red aggregates in intact mitochondria, while green fluorescence is due to the formation of JC-1 monomers at low mitochondrial membrane potential. As shown in Fig. 3A, lidocaine and bupivacaine significantly increased the formation of JC-1 monomers in 8505C and K1 cells in a dose-dependent way. The percentage of cells with low ΔΨ_m_ was slightly lower than the percentage of apoptotic cells treated with local anesthetics at the same concentrations.

We next sought to determine whether cytochrome c was released from mitochondria to the cytosol. As expected, a higher level of cytochrome c was measured in cytosol in both cell lines after treatment with lidocaine and bupivacaine (Fig. 3B). Collectively, these data suggest that treatment of thyroid cancer cells with local anesthetics leads to activation of the mitochondrial apoptotic pathway.

### Activation of caspases by local anesthetics

**Figure 4:**
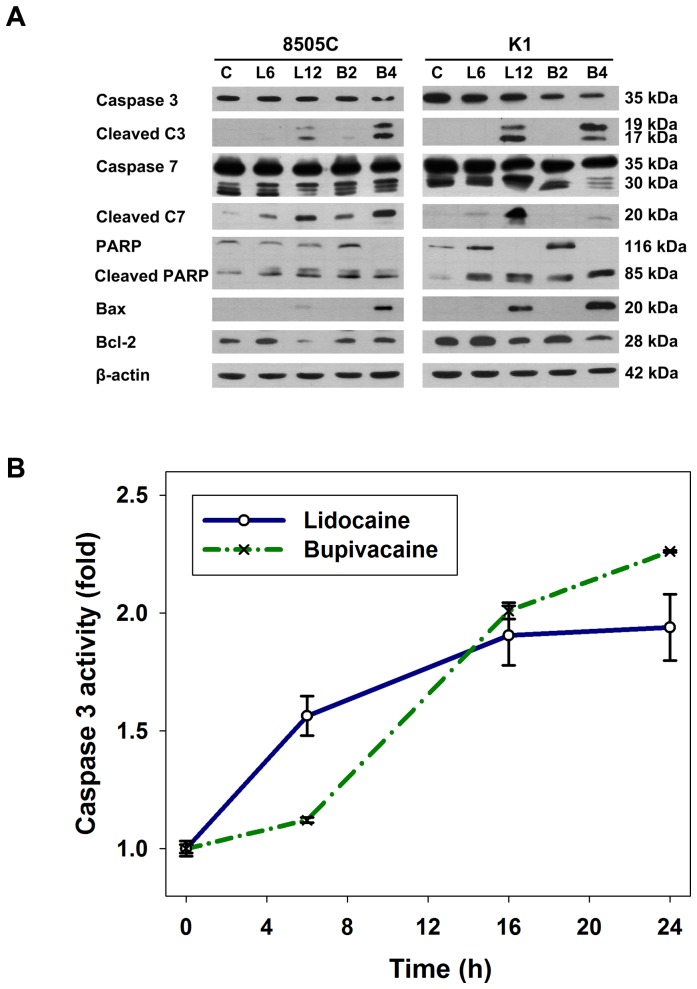
Expression of apoptotic proteins and caspase 3 activities in human thyroid cancer cells treated with local anesthetics. (**A**) 8505C and K1 cells were treated with the indicated concentrations of lidocaine (L6, 6 mM; L12, 12 mM) and bupivacaine (B2, 2 mM; B4, 4 mM) for 16 h. Cells were harvested and samples were prepared for Western blot analysis. C, control. PARP, poly(ADP-ribose) polymerase. (**B**) Activity of caspase 3 in cell lysates from 8505C cells treated with lidocaine (12 mM) and bupivacaine (4 mM) for different time periods was determined using a colorimetric protease assay. Error bars represent standard error of the mean.

Release of cytochrome c has been shown to activate the downstream caspases that are ultimately required to induce apoptosis. We therefore examined whether caspase activation was involved in the induction of apoptosis by local anesthetics. Treatment with lidocaine and bupivacaine resulted in the activation of caspase 3 and caspase 7, and cleavage of PARP in 8505C and K1 cells dose-dependently (Fig. 4A). Furthermore, caspase colorimetric substrate assay showed that caspase 3 activity increased in a time-dependent manner in 8505C cells after treatment with lidocaine and bupivacaine (Fig. 4B). These results clearly indicate that caspase activation plays an important role in thyroid cancer cell apoptosis induced by local anesthetics.

The proteins of the Bcl-2 family participate in the apoptotic process by functioning as promoters (*e.g.*, Bax) or inhibitors (*e.g.*, Bcl-2). To activate the mitochondrial apoptotic pathway, activated Bax forms an oligomeric pore and results in the permeabilization of the mitochondrial outer membrane. We found that treatment with lidocaine and bupivacaine lead to a reduction in the Bcl-2 levels with a concomitant increase in the Bax levels (Fig. 4A). Therefore, local anesthetics alter the protein levels of key members of the Bcl-2 family in a manner that favors an increase in the ratio of Bax/Bcl-2, which may contribute to the susceptibility of thyroid cancer cells to apoptosis.

### Analysis of gene expression signatures affected by local anesthetics

To identify gene expression signatures that are associated with biological functions of local anesthetics, microarray and pathway enrichment analysis was carried out to compare expression patterns in 8505C cells treated with lidocaine and bupivacaine. The top ten pathways identified by the pathway enrichment analysis are listed in Tables 1 and 2. It is noteworthy that the most prominent transcriptional change in thyroid cancer cells treated with local anesthetics is apoptosis. Other pathways common to lidocaine and bupivacaine treatment include cytoskeletal remodeling and myeloid differentiation. Furthermore, we used *in silico* tools from Ingenuity to identify pathways reportedly involving molecular mechanism of cancer. Integrating microarray data from cells treated with lidocaine and bupivacaine, pathway analysis suggested that extracellular signal-regulated kinase (ERK) 1/2 and p38 MAPK are significantly up- or down-regulated in response to treatment with local anesthetics (Fig. S2).

**Table 1:** Top ten pathways identified by pathway enrichment analysis of differentially expressed genes of 8505C human thyroid cancer cells following treatment with lidocaine (12 mM) for 24 hours.

Pathway	P value	FDR
Cytoskeleton remodeling_TGF, WNT and cytoskeletal remodeling	8.467E-10	5.92707E-07
Apoptosis and survival_FAS signaling cascades	5.344E-08	1.87052E-05
Regulation of lipid metabolism_RXR-dependent regulation of lipid metabolism via PPAR, RAR and VDR	1.674E-07	3.90509E-05
Blood coagulation_Blood coagulation	1.958E-07	0.000129219
Development_Hedgehog signaling	7.555E-07	0.000132204
Development_Role of HDAC and calcium/calmodulin-dependent kinase (CaMK) in control of skeletal myogenesis	1.032E-06	0.000144549
Development_G-CSF-induced myeloid differentiation	1.316E-06	0.00043431
Neurophysiological process_Receptor-mediated axon growth repulsion	2.335E-06	0.00024149
Signal transduction_cAMP signaling	2.415E-06	0.00024149
Immune response_HMGB1/RAGE signaling pathway	2.982E-06	0.000252541

FDR, false discovery rate.

**Table 2:** Top ten pathways identified by pathway enrichment analysis of differentially expressed genes of 8505C human thyroid cancer cells following treatment with bupivacaine (4 mM) for 24 hours.

Pathway	P value	FDR
Protein folding and maturation_POMC processing	1.843E-10	1.2825E-07
Immune response_IL-17 signaling pathways	2.347E-07	0.000101697
Development_G-CSF-induced myeloid differentiation	3.077E-07	0.000101697
Protein folding and maturation_Bradykinin/Kallidin maturation	7.610E-07	0.000167678
DNA damage_Role of Brca1 and Brca2 in DNA repair	1.150E-06	0.000400323
Immune response_IFN alpha/beta signaling pathway	1.874E-06	0.000434714
Apoptosis and survival_FAS signaling cascades	3.884E-06	0.000675776
Immune response_MIF-mediated glucocorticoid regulation	4.375E-06	0.000722955
Protein folding and maturation_Angiotensin system maturation\Human version	6.366E-06	0.000841555
Cytoskeleton remodeling_TGF, WNT and cytoskeletal remodeling	8.117E-06	0.000953231

FDR, false discovery rate.

### Modulation of mitogen-activated protein kinase signaling

**Figure 5:**
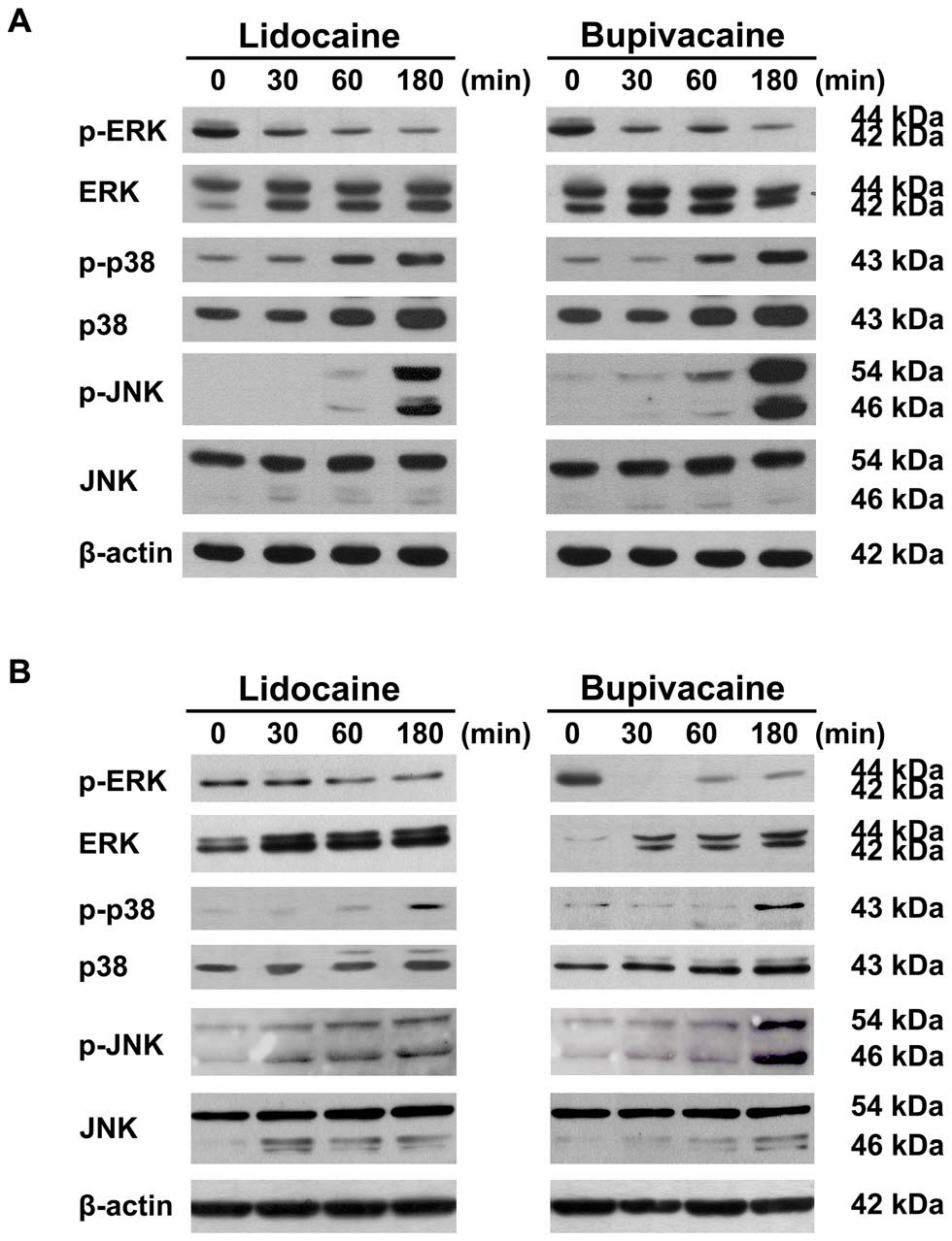
Mitogen-activated protein kinase signaling in human thyroid cancer cells treated with local anesthetics. 8505C (**A**) and K1 (**B**) cells were treated with lidocaine (12 mM) and bupivacaine (4 mM) for various periods of time, and activities of ERK, p38, and JNK were examined by Western blot analysis using phospho-specific antibodies. The total protein levels of ERK, p38, and JNK were also measured.

Our microarray and pathway enrichment analysis indicates that MAPK pathways are probably involved in the mechanisms mediating action of local anesthetics on thyroid cancer cells. To examine this possible relationship, total cellular proteins were extracted from 8505C and K1 cells treated with lidocaine (12 mM) and bupivacaine (4 mM) for various time periods, and lysates were immunoblotted with specific antibodies against phosphorylated and total ERK1/2, p38, and c-Jun N-terminal kinase (JNK), respectively. As shown in Fig. 5, a decreased phosphorylation of ERK1/2 was observed. Lidocaine and bupivacaine significantly increased phosphorylation of p38 and JNK. Lidocaine and bupivacaine had no remarkable effect on total p38 and JNK protein level.

### Effects of inhibition of mitogen-activated protein kinases

**Figure 6:**
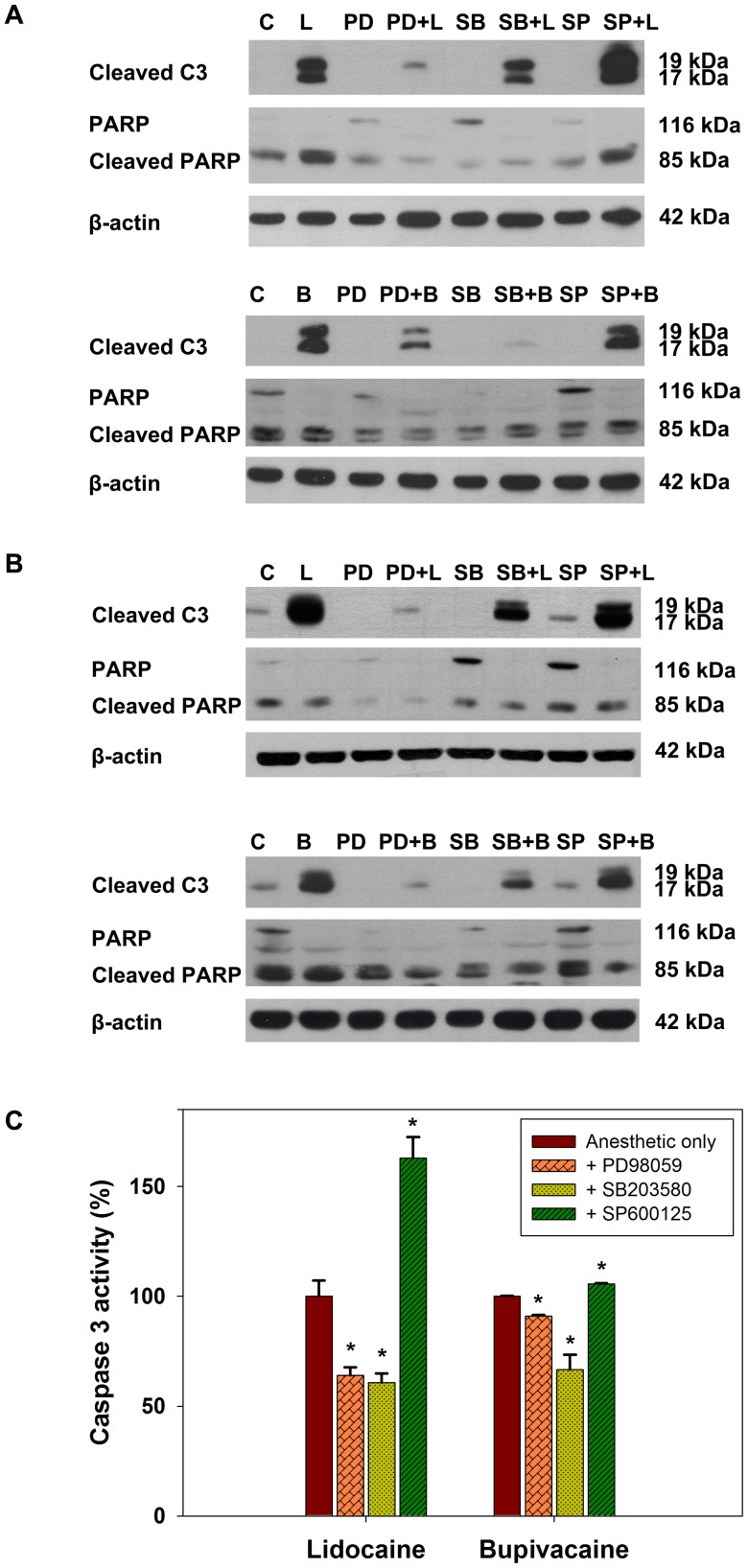
Effects of inhibition of mitogen-activated protein kinase signaling on apoptosis in human thyroid cancer cells treated with local anesthetics. 8505C (**A**) and K1 (**B**) cells were cotreated with 30 μM PD98059 (PD), 30 μM SB203580 (SB), or 30 μM SP600125 (SP) in addition to 12 mM lidocaine (L) or 4 mM bupivacaine (B) for 16 h. Cells were harvested and samples were prepared for Western blot analysis. C, control. C3, caspase 3. PARP, poly(ADP-ribose) polymerase. (**C**) Activity of caspase 3 in cell lysates from 8505C cells treated with lidocaine (12 mM) and bupivacaine (4 mM) with or without specific inhibitors for 24 h was determined using a colorimetric protease assay. Error bars represent standard error of the mean. *, P<0.01 *versus* control.

Subsequently, we would like to know whether the modulations of ERK1/2, p38 MAPK, and JNK signaling pathways account for apoptosis induced by local anesthetics. To this end, we examined the effects of specific inhibitors on caspase activation and PARP cleavage. PD98059 is an inhibitor of MAPK/ERK kinase (MEK), thereby inhibiting the phosphorylation and the activation of MAPK. SB203580 is a selective, reversible, and ATP-competitive inhibitor of p38 MAPK, whereas SP600125 is an anthrapyrazolone JNK inhibitor that competes with ATP to inhibit the phosphorylation of c-Jun. As shown in Figs. 6A and 6B, PD98059 and SB203580, but not SP600125, reduced the activation of caspase 3 and cleavage of PARP. These results were confirmed by caspase 3 activity assay (Fig. 6C). Cotreatment with PD98059 or SB203580 suppressed caspase 3 activity, whereas SP600125 paradoxically increased caspase 3 activity following treatment with local anesthetics. These data are accordant with the results of pathway enrichment analysis (Fig. S2) that ERK1/2 and p38 MAPK signaling pathways are involved in apoptosis induced by local anesthetics.

## Discussion

It has been more than a century since the first synthetic local anesthetic was introduced in the early 1900s. Local anesthetics bind reversibly to a specific receptor site within the sodium channels and block ion movement. It was believed that the effects are reversible with recovery of nerve function and without damage to neuronal cells. However, reports of permanent neurologic injury have generated concern about the neurotoxicity of local anesthetics [18]. Accumulating evidence suggests that local anesthetics can cause rapid neuronal death through triggering apoptosis and necrosis [19], [20]. Moreover, a number of studies have reported local anesthetic toxicity on other cell types [21]–[23]. In the present study, we firstly demonstrated the direct effect of lidocaine and bupivacaine inhibiting cell growth and colony formation of thyroid cancer cells.

The mechanisms of cell toxicity from local anesthetics have not been fully elucidated. In U937 histiocytic lymphoma cells, lidocaine induced apoptosis at concentrations below 12 mM and induced necrosis at concentrations above 15 mM [24]. Similar observations have been reported by others [19], [20], [25]. In this study, both apoptosis and necrosis participate in cell death induced by local anesthetics. Intriguingly, a slight antagonism was identified with combination of lidocaine and bupivacaine. It seems likely that lidocaine and bupivacaine induce cell death by a similar mechanism. When a cell is unable to die by apoptosis, it may undergo necrotic cell death. It is well known that ATP levels are a determinant of manifestation of cell death [26], [27]. Lidocaine and bupivacaine were shown to decrease ATP levels and viability of melanoma cells [28]. The mode of cell death likely depends on the duration of exposure and concentrations of local anesthetics.

Mitochondrial energetics affected by local anesthetics may account for the decrease in the ATP levels. Local anesthetics can reach mitochondria and this effect is highly dependent on the lipid-solubility of the local anesthetic [29]. Clinical and experimental myopathy induced by bupivacaine has been reported to result from mitochondrial dysfunction and reduced oxidative energy metabolism [30], [31]. Specifically, bupivacaine suppressed cell respiration through inhibition of complexes I and III, accompanied with production of reactive oxygen species [32]. Irwin *et al.* have shown that bupivacaine myotoxicity relied on active oxidative metabolism because highly glycolytic extensor digitorum longus were resistant to bupivacaine-induced toxicity [30]. Nonetheless, tumor cells predominantly produce energy through glycolysis (Warburg effect) [33]. Our findings are in agreement with those of a few reports demonstrating that mitochondrial dysfunction could also be elicited by local anesthetics in malignant tumor cells [24], [25], [34].

Mitochondria are essential modulators of apoptosis, necrosis and autophagy [27]. The intrinsic (mitochondrial) pathway of apoptosis is mediated by the release of pro-, and anti-apoptotic proteins, including cytochrome c. In our experiments, local anesthetics increased pro-apoptotic Bax expression and downregulated Bcl-2 expression, leading to a higher ratio between pro-apoptotic and anti-apoptotic Bcl-2 family proteins. That increased Bax/Bcl-2 ratio facilitates the release of mitochondrial cytochrome c, which subsequently induces activations of caspase cascade that cleave PARP into inactive fragments. However, lidocaine and bupivacaine promoted mitochondrial injury to an extent less than that of apoptosis induced by the same concentrations of the local anesthetic. This suggests that a mitochondria-independent process may also play a role. This hypothesis is supported by the results of pathway enrichment analysis (Fig. S2), in which transcriptional alterations in the death receptor pathway components were involved.

Cellular microarrays are powerful experimental tools for identification of novel drug targets and pharmacogenomics. Unami and colleagues firstly reported that apoptosis-related genes were transcriptionally regulated by bupivacaine according to microarray analysis [34]. Recently, Lucchinetti *et al.* demonstrated that multiple transcriptional programs related to cell differentiation, tumorigenesis, and metastasis were negatively affected by ropivacaine [35]. The authors postulate that this means novel rationales for the perioperative use of local anesthetics in patients with cancer. Our data are in concordance with these results and showed a strong association between local anesthetics-induced alterations in gene expression and apoptosis. Furthermore, we identified that MAPK pathway is involved in the molecular mechanisms of apoptosis induced by local anesthetics.

In primary neuron cultures, bupivacaine and ropivacaine were found to activate p38 MAPK and JNK but not ERK1/2 [16]. Furthermore, pharmacological inhibition of these kinases attenuates neurotoxicity *in vitro*. The authors noted that axonal degeneration induced by lidocaine is prevented by p38 MAPK inhibitor but not by caspase inhibition [36]. Harato *et al.* also demonstrated that p38 MAPK inhibitor treatment attenuated the caspase 3 activity and cell death induced by bupivacaine [37]. In another study, p38 MAPK pathway is implicated in bupivacaine-induced apoptosis in human neuroblastoma SH-SY5Y cells [17]. In line with the findings in neuronal apoptosis, we found that p38 MAPK activation plays a crucial role in local anesthetics-induced apoptosis in thyroid cancer cells. Negative regulation of proliferation is a highly conserved and important function of p38α in various cell types [38]. Interestingly, upregulated p38α activity has been reported in papillary and follicular thyroid cancer [39]. The notion is supported by the basal expression of phospho-p38 proteins in this study (Fig. 5). The dual role for p38 MAPK in thyroid cancer biology remains a virtually untapped area in spite of Ras-Raf-ERK pathway having been extensively studied in thyroid cancer.

In the present study, ERK1/2 activity was suppressed by lidocaine and bupivacaine. Joo *et al.* demonstrated that lidocaine significantly suppressed the increased ERK1/2 response in a rat model of neuropathic pain [40]. More recently, lithium was shown to protect against bupivacaine-induced neuronal injury through reversing the suppression of ERK1/2 signaling [41]. There is increasing evidence for a crosstalk between MAPK pathways. For example, p38 and JNK pathway activation in the induction of apoptosis may negatively regulate the ERK pathway [42]. It is also worth noting that there is a paradoxical decrease in caspase activation following PD98059 treatment. Many genetic alterations have been implicated in thyroid cancer, and the aberrant activation of the Ras-Raf-ERK pathway is most frequent [43]. Inhibition of the MAPK pathway arrests thyroid cancer cells in G1 phase [44]. Therefore, a plausible explanation for our results is that ERK1/2 inhibition increased susceptibility of thyroid cancer cells to other mechanisms of growth inhibition like cell cycle arrest and necrosis, which do not involve caspase activation.

## Conclusions

In summary, our study deciphered the molecular mechanisms responsible for the cytotoxicity induced by local anesthetics in thyroid cancer cells, presenting the involvement of MPAK signaling pathways and suggesting potential benefits of the use of local anesthetics in clinical practice.

## Materials and Methods

### Cell culture and reagents

The human thyroid carcinoma cell lines 8505C and K1 were purchased from the German Collection of Microorganisms and Cell Cultures (DSMZ, Braunschweig, Germany) and the European Collection of Cell Cultures (ECACC, Salisbury, United Kingdom), respectively. 8505C cells were cultured at 37°C in a humidified atmosphere of 5% CO_2_ in RPMI 1640 medium (Invitrogen/Gibco, Carlsbad, CA) supplemented with 10% fetal bovine serum (FBS). K1 cells were cultured in Dulbecco's modified Eagle's medium (Gibco) mixed with Ham's F12 (Gibco) and MCDB 105 (Sigma, St. Louis, MO) medium in 2∶1∶1 proportions, supplemented with 10% FBS and 2 mM L-glutamine. Both 8505C and K1 have been authenticated to be unique thyroid cancer cell lines [45]. Lidocaine, bupivacaine, procaine, PD98059, SB203580, and SP600125 were obtained from Sigma. Osmolality of the culture media for experiments was analyzed by the freezing point depression method using an Advanced 3320 Micro Osmometer (Advanced Instruments, Norwood, MA).

### Cell viability assay and drug combination analysis

Cell viability assay was performed in triplicate for each experiment as previously described [46]. Briefly, 8505C and K1 cells were seeded into 96-well plates one day before lidocaine and bupivacaine (individually or in combinations) was added at the indicated concentrations. For the thiazolyl blue tetrazolium bromide (MTT) colorimetric assay, 100 µl of MTT reagent (5 mg/ml; Sigma) was added to the cell culture and cells were incubated at 37°C for 4 h. The formazan crystals converted from tetrazolium salts by viable cells were solubilized with acidified isopropanol. The absorbance was measured with Varioskan Flash (Thermo Fisher Scientific, Waltham, MA) at wavelength of 570 nm. The absorbance of control cells (incubated without drugs) was defined as 100%.

Drug synergy was determined by the CI and isobologram analyses according to the median effect methods described by Chou and Talalay [47]. On the basis of the dose-response curves from MTT assays, the CI values were calculated. Synergism, additivity and antagonism are defined as CI <1, CI  = 1 and CI >1, respectively. In the isobologram graph, combination data points that fall on, above, and beneath the oblique line represent an additive, antagonistic, and synergistic effect, respectively.

### Colony formation assay

For colony formation assay, 400 cells/well were seeded into six-well plates, allowed to adhere for 24 h, and treated with the indicated concentrations of lidocaine and bupivacaine from day 2. After 8 to 15 days, colonies were stained with 3% crystal violet and colonies containing >50 cells were counted.

### Cell cycle analysis

The effect of lidocaine and bupivacaine on the cell cycle was analyzed by PI staining and flow cytometry [48]. After treatment with lidocaine and bupivacaine for 6, 24, and 48 h, thyroid cancer cells were harvested, gently washed, and fixed in 70% cold ethanol at 4°C overnight. The fixed cells (1×10^6^) were incubated with RNase A for 30 min at room temperature, and stained with PI solution using the BD Cycletest Plus DNA reagent kit (BD Biosciences, San Jose, CA) in the dark. Subsequently, the cells were analyzed on a FASCalibur flow cytometer (BD Biosciences) equipped with CellQuest Pro software. The distribution of cells in G0/G1, S and G2/M phases of cell cycle was estimated using the ModFit LT software (Verity Software House, Topsham, ME). As an estimate of the proportion of apoptotic cells, the percentage of hypodiploid cells was calculated in the DNA histogram.

### Analysis of cell apoptosis

Cell apoptosis was analyzed using the annexin V-fluorescein isothiocyanate (FITC) apoptosis detection kit (BD Biosciences Pharmingen, San Diego, CA) according to the instructions of the manufacturer. Thyroid cancer cells were treated with the indicated concentrations of lidocaine and bupivacaine for 24 to 72 h. Both floating and attached cells were harvested and washed, followed by incubation with 5 μl of annexin V-FITC and 5 μl of PI for 15 min at room temperature in the dark. After the incubation, 400 μl of binding buffer solution was added, and flow cytometry analysis was performed.

### Determination of mitochondrial membrane potential (ΔΨ_m_)

Mitochondrial membrane potential was determined by flow cytometry using the ΔΨ_m_-dependent fluorescent dye JC-1 (CS0390; Sigma). JC-1 is a lipophilic, cationic dye that that can selectively enter into mitochondria, and undergoes a reversible change in fluorescence emission according to the ΔΨ_m_. Healthy cells with high ΔΨ_m_ will form JC-1 aggregates and fluoresce red, whereas those apoptotic cells with low ΔΨ_m_ will contain monomeric JC-1 and fluoresce green. After treatment with lidocaine and bupivacaine for the indicated time, thyroid cancer cells were harvested and incubated with JC-1 for 20 min at 37°C according to the manufacturer's instructions. The samples were then subject to flow cytometry.

### Western blot analysis

Total cellular proteins were extracted, quantified, and subjected to gel electrophoresis according to standard procedures as we described previously [49]. The antibodies used in our study, including anti-caspase 3 (#9662), anti-cleaved caspase 3 (#9661), anti-caspase 7 (#9494), anti-poly(ADP-ribose) polymerase (PARP) (P248; Sigma), anti-Bax (#2774), anti-Bcl-2 (#2872), anti-phospho-ERK (#9101), anti-ERK (#9102), anti-phospho-p38 (#4511), anti-p38 (#9212), anti-phospho-JNK (#9255), anti-JNK (#9252), and anti-actin (A5441; Sigma). Antibodies were obtained from Cell Signaling, Danvers, MA unless specified otherwise. The antigen-antibody complexes were visualized with by chemiluminescence with the Amersham ECL detection system (GE Healthcare, Piscataway, NJ).

### Measurement of cytochrome c release

To determine the release of cytochrome c from mitochondria to cytoplasm, preparations of cytosolic extracts were carried out with the Mitochondria Isolation Kit (#89874; Thermo Scientific/Pierce, Rockford, IL) according to the manufacturer's instructions. In brief, thyroid cancer cells (2×10^7^) were harvested after treatment with lidocaine and bupivacaine for the indicated time periods, washed, and incubated with cytosol extraction buffer in ice for 10 min. The supernatant was collected through centrifugation at 700×g for 10 min at 4°C. The cytosolic fraction was obtained through centrifugation again at 12,000×g for 15 min at 4°C, and was analyzed by Western blotting using anti-cytochrome c antibody (#4272; Cell Signaling) as described above.

### Assay for caspase 3 activity

The caspase 3 activity was assayed using the ApoTarget Caspase-3 Colorimetric Protease Assay Kit (KHZ0022; Invitrogen) according to the manufacturer's protocol. Briefly, 5×10^6^ thyroid cancer cells were treated with lidocaine and bupivacaine with or without specific inhibitors for the indicated time periods. The cells were harvested using trypsinization and cell lysates prepared as described above. Samples of the cell lysates (100 μg protein per sample) were mixed with reaction buffer and 200 μM substrate (DEVD-*p*NA) and incubated for 2 hours at 37°C in the dark. The absorbance was then measured at 405 nm and the sample readings calculated by subtracting the absorbance of blank samples.

### Global gene expression analysis

8505C cells were treated with lidocaine (12 mM), bupivacaine (4 mM), or left untreated for 24 hours. Cells were harvested and total RNA was isolated using the TRI Reagent (Sigma). After RNA integrity was verified, cDNA was synthesized using the Superscript Double-Stranded cDNA Synthesis Kit (Invitrogen), subsequently labeled using the One-Color DNA Labeling Kit (Roche NimbleGen, Madison, WI), and hybridized to a Human HG18 expression array (12×135K) using the NimbleGen Hybridization System. Arrays were scanned and chip images were collected on a NimbleGen MS200 Microarray Scanner. Following the acquisition and initial quantification of array images, raw array data were normalized per chip and per gene and filtered based on raw signal intensity and detection call. Genes with an expression fold change of ≥2 between a treatment and a control were considered to be significant. To determine the potential mechanistic network, transcripts with differential expression were studied using the MetaCore pathway analysis suite (GeneGo-Thomson Reuters, New York, NY) and Ingenuity Pathway Analysis (Ingenuity Systems, Redwood City, CA).

### Statistical analysis

Results were expressed as means ± SEM. Comparisons between groups were performed using a two-tailed Student's t test. Values of P<0.05 were considered significant.

## Supporting Information

Figure S1 **Final pH and osmolality of the culture media for experiments.**
(TIF)

Figure S2 **Molecular mechanism of cancer from Ingenuity Pathways Analysis.**
(TIF)
