# Supplementary figures and images for: Local Anesthetics Induce Apoptosis in Human Thyroid Cancer Cells through the Mitogen-Activated Protein Kinase Pathway
Source: PLoS One. 2014 Feb 21;9(2):e89563. doi: 10.1371/journal.pone.0089563 (PMC3931808; doi:10.1371/journal.pone.0089563)

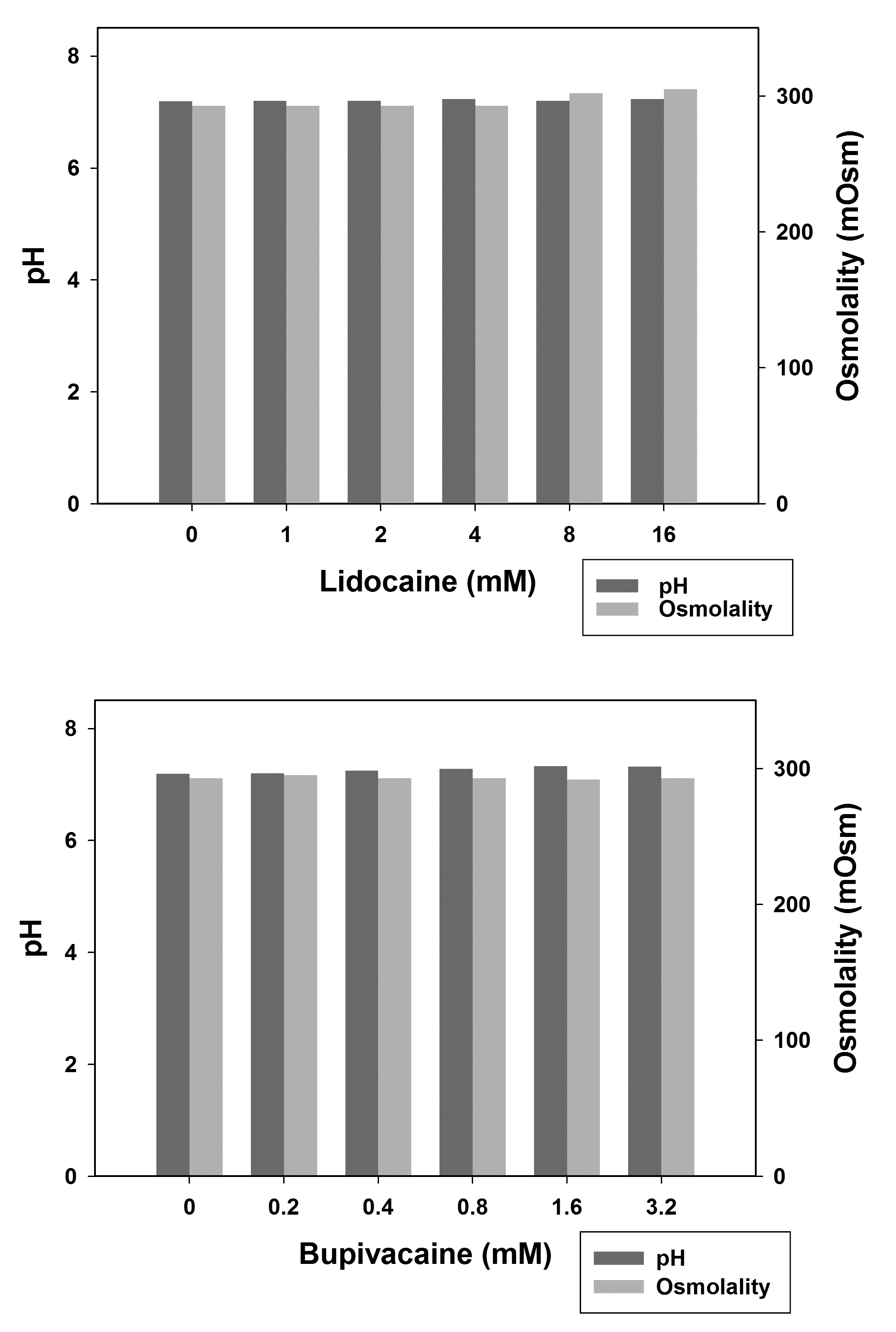

Supplement: Figure S1 — Final pH and osmolality of the culture media for experiments. (TIF) [file pone.0089563.s001.tif]

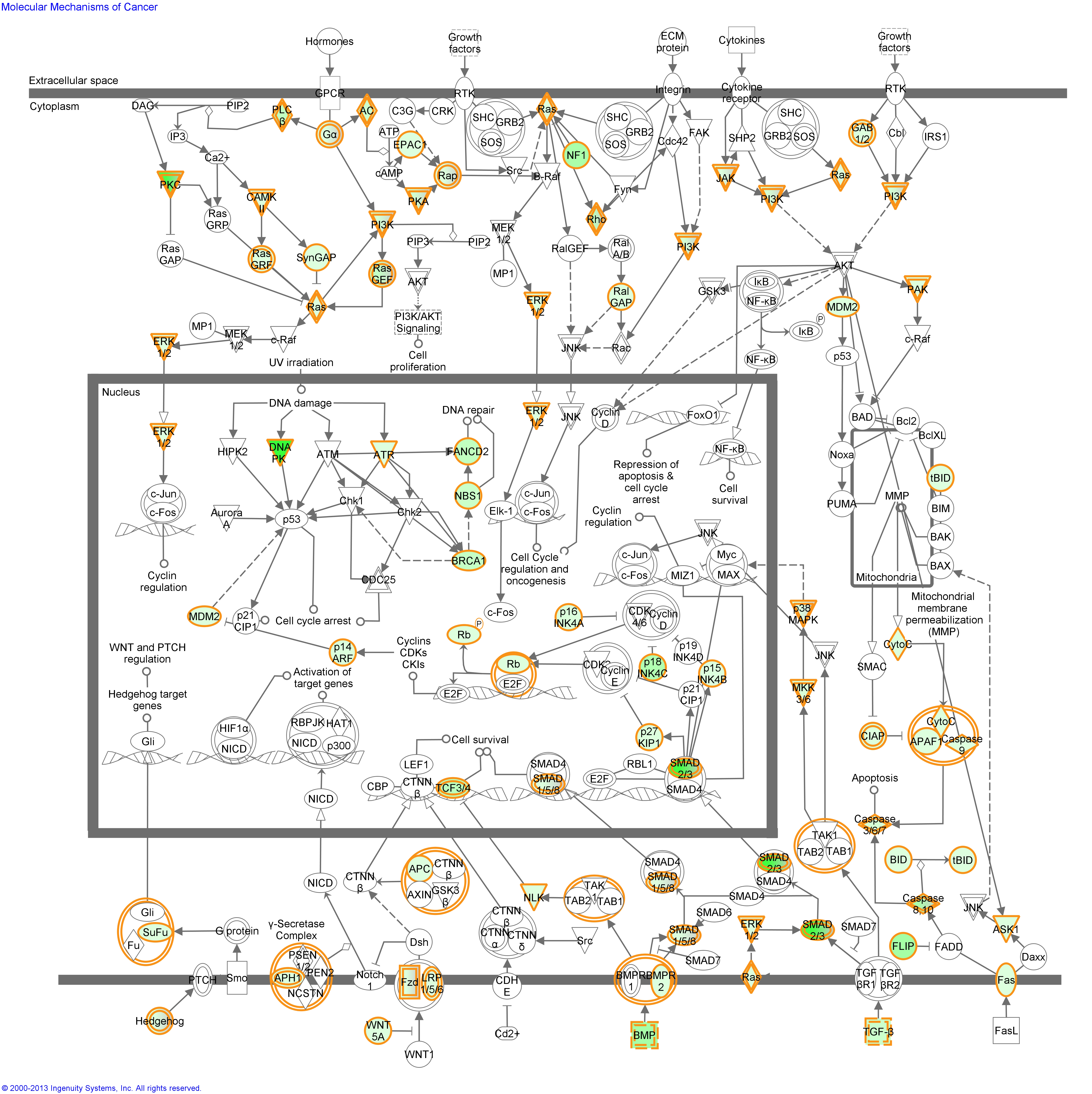

Supplement: Figure S2 — Molecular mechanism of cancer from Ingenuity Pathways Analysis. (TIF) [file pone.0089563.s002.tif]
